# Advances in Drug Design and Development for Human Therapeutics Using Artificial Intelligence—I

**DOI:** 10.3390/biom12121846

**Published:** 2022-12-10

**Authors:** Dongqing Wei, Gilles H. Peslherbe, Gurudeeban Selvaraj, Yanjing Wang

**Affiliations:** 1 Department of Bioinformatics, The State Key Laboratory of Microbial Metabolism, College of Life Sciences and Biotechnology, Shanghai Jiao Tong University, Shanghai 200240, China; 2 Centre for Research in Molecular Modeling (CERMM) & Department of Chemistry and Biochemistry, Concordia University, Montreal, QC H4B 1R6, Canada

Artificial intelligence (AI) has emerged as a key player in modern healthcare, especially in the pharmaceutical industry for the development of new drugs and vaccine candidates. AI technology has shown promising results in target identification, protein–protein interactions, protein–drug interactions, metabolic pathway identification, drug repurposing, facilitating de novo drug design to combat diseases, and increasing short-term research productivity. This Special Issue highlights recent advances in computational modeling and dynamics simulations for elucidating phosphodiesterase 9 (PDE9) inhibitors with different metal systems; the identification of potential inhibitors of coronavirus disease 2019 (COVID-19); the prediction of convolutional network-based drug–target interaction modules; the building of transformer-based deep neural networks for metabolomics; and the development of autoencoder-based deep learning models for drug repurposing for Alzheimer’s disease. 

Signal transduction systems such as cyclic adenosine monophosphate (cAMP) and cyclic guanosine monophosphate (cGMP) regulate cellular functions in organisms, and the dysregulation of these signaling pathways leads to various diseases. To minimize the dysregulation of signaling pathways and the disruption of cellular function, Sivakumar et al. [1] targeted and studied PDE9 inhibitors with different metal systems. PDE and its isoforms are among the key enzymes that hydrolyze the cAMP and cGMP signaling pathways. Moreover, many studies have shown that metal-containing (e.g., Zn/Mg/Mn) enzymes are involved in the pathophysiology and etiology of human diseases ranging from infectious diseases to cancer. The study by Sivakumar et al. provides new insights into the interaction patterns between inhibitors and metalloenzymes.

The repurposing of ‘existing’ drugs to treat common and emerging diseases increasingly involves the targeting of lower-risk compounds and results in reduced development costs and times. The COVID-19 pandemic has disrupted healthcare systems globally since 2019, calling for new therapeutics that target severe acute respiratory syndrome coronavirus 2 (SARS-CoV-2), which is responsible for the disease. Antiviral treatments have been known to reduce disease spread, infection, and severity for over 30 years. Mohammad et al. [2] studied the inhibitory effect of the known antiviral drug candidate remdesivir on the RNA-dependent RNA polymerase protein (RdRp) of SARS-CoV-2. The core component—the RdRp complex—and its catalytic subunit play critical roles in the viral replication cycle, and thus, agents that target the RdRp complex inhibit replication of the virus. The authors’ computational findings support the repurposing of remdesivir to target RdRp in wild-type and mutant SARS-CoV-2. 

The prediction and identification of compound–protein interactions (CPIs) play an important role in the recent discovery and development of safe drug candidates. Many studies point to the advantages of deep learning models for learning task-related functions from large datasets on compound–protein interactions. Wang et al. [3] developed a multiscale convolutional network (MCN) model for CPI prediction. This model extracts the local and global features of proteins and the topological features of compounds and yields superior predictions to existing deep learning methods.

In the past decades, high-throughput screening (HTS) experiments have greatly accelerated the identification of drug–target interactions (DTIs). However, HTS experiments are costly and cumbersome, and cannot meet the need to reveal DTIs for millions of existing drug and target combinations. Jin et al. [4] developed a new DTI model called EmbedDTI to improve the representation of both drug and target proteins as well as the performance of DTI prediction. The use of machine learning techniques in DTIs is limited due to the difficulty of extracting useful information from drug and target property libraries. The authors used protein language modeling to pre-train amino acid feature embedding and used a ligand atom graph, a substructure graph, and an adaptation graph to train a convolutional neural network learning model. The authors benchmarked their model and concluded that EmbedDTI is superior to other DTI predictors.

Mass spectrometry (MS) fingerprinting is key to metabolomics (i.e., measurements of small molecules in biological matrices). The identification of metabolite molecules is currently performed primarily by comparing MS data with those of a limited number of authentic chemical standard libraries. Only 10% of molecules can typically be identified experimentally from reproducible spectral features of complex matrices (e.g., serum), despite many heuristics (such as the loss of neutral masses, and isotope patterns). To address the above issues, Shrivastava et al. [5] developed MassGenie, a method that uses transformer-based deep neural networks. Using this approach, the effective properties of mass spectral fragments and valence space can be learned to generate candidate molecular structures that are very similar/identical to those of the “real” molecule. Furthermore, MassGenie can generate and “learn” millions of fragmentation patterns in-computer and predict candidate structures directly from mass spectra, opening up an avenue for de novo small molecule structure prediction from experimental MS.

Finally, Chyr et al. [6] proposed a deep learning model called DOTA (Drug Repositioning Approach Using Optimal Transport for Alzheimer’s disease). Alzheimer’s disease is the leading cause of age-related dementia, affecting over five million people in developing countries and leading to significant healthcare costs worldwide. Unfortunately, current treatments can reduce and/or control cognitive symptoms at best, but do not provide a cure for the disease. Using multimodal autoencoders and Wasserstein variational autoencoders to target key biological pathways, DOTA promises to improve patient cognition, circadian rhythms, and Alzheimer’s disease pathogenesis, and to predict the repurposing of potential drug candidates.

We hope that the interdisciplinary topics covered in this collection will prompt further discussion among researchers and promote innovation in the field of artificial intelligence for drug design and development. Above all, we would like to thank the authors for their excellent contributions. We also thank the scientific experts in AI, computational modeling, and drug design who offered invaluable comments and suggestions to improve the quality of this Special Issue. Finally, we would like to thank the Editor-in-Chief, Section Managing Editor, and all the editorial staff of MDPI—*Biomolecules* for the opportunity to work with them on this enlightening editorial journey.
